# Supplementary material for: Identification of photoperiod-induced specific miRNAs in the adrenal glands of Sunite sheep (Ovis aries)
Source: Front Vet Sci. 2022 Jul 22;9:888207. doi: 10.3389/fvets.2022.888207 (PMC9354845; doi:10.3389/fvets.2022.888207)
Supplement: Supplementary file 1 [file Data_Sheet_1.ZIP › Supplementary file/miRDeep2 file.zip!/miRDeep2 file/Know_Structure/SP42A3/oar-mir-30c.pdf]

miRBase precursor : oar-mir-30c
Total read count : 108977
oar-miR-30c read count : 108810
remaining reads : 167

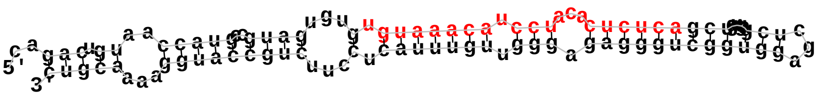

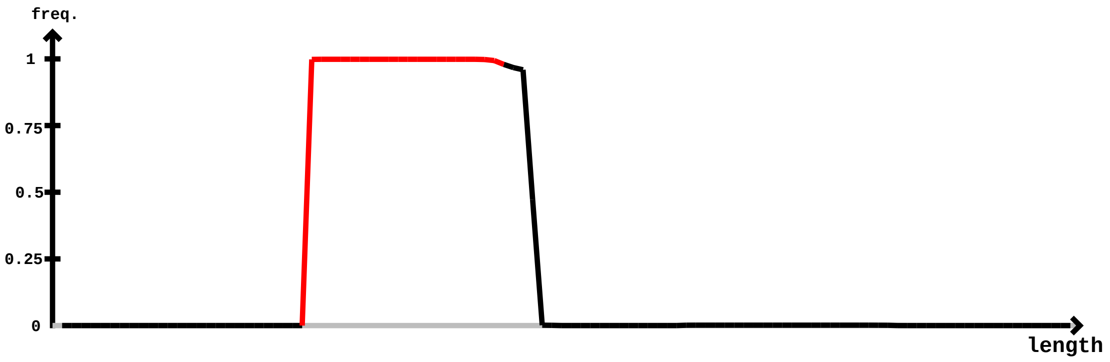


```
                                        oar-miR-30c
5'- cagacuguaaccaugccguagugugunguaaacauccuacacucucagcugugagcucgagguggcugggagaggguuguuuacuccuucugccauggaaaacguc   -3'    exp
```

oar-miR-30c			
5'- cagacuguaaccaugccguagugug**uguaaacauccuacacucuca**gcugugagcucgagguggcugggagaggguuguuuacuccuucugccauggaaaacguc -3'	exp		
..(((.((..(((((..((((...(.(((((((.(((...((((((((....(((...))))))))))).))).))))))).)...)))))))))...)))))	reads	mm	sample
cagacuguaaccaugccguagugu..	1	0	seq
....cuguaaccaugccguagugu..	1	0	seq
....cuguaaccaugccguagugug...	2	0	seq
........................uguaaacauccuacacuU..	22	1	seq
........................uguaaacauccuacacuc..	72	0	seq
........................uguaaacaCccuacacuc..	1	1	seq
........................uguGaacauccuacacuc..	1	1	seq
........................uguaaacauccuacacucu...	373	0	seq
........................uguaaacaCccuacacucu...	45	1	seq
........................uguGaacauccuacacucu...	3	1	seq
........................uguaaacauccuacacuUu...	1	1	seq
........................uguaaacauccuacacucuc..	1451	0	seq
........................uguaaacaCccuacacucuc..	136	1	seq
........................uguaaacaCccuacacucuca...	128	1	seq
........................uguaaacauccuacacucuca...	1160	0	seq
........................uguaaacaCccuacacucucag..	97	1	seq
........................uguaaacauccuacacucucag..	800	0	seq
........................uguaaacaCccuacacucucagc...	4509	1	seq
........................uguaaacauccuacacucucagc...	48532	0	seq
........................uguaaacaCccuacacucucagcu..	3981	1	seq
........................uguaaacauccuacacucucagcu..	47297	0	seq
........................uguaaacaCccuacacucucagcug...	2	1	seq
........................uguaaacauccuacacucucagcug...	57	0	seq
........................uguaaacauccuacacucucagcugu..	90	0	seq
........................uguaaacaCccuacacucucagcugu..	4	1	seq
........................uguaaacaCccuacacucucagcugug...	1	1	seq
.........................guaaacauccuacacucu...	1	0	seq
.........................guaaacauccuacacucuca...	1	0	seq
.........................guaaacauccuacacucucagc...	16	0	seq
.........................guaaacaCccuacacucucagc...	1	1	seq
.........................guaaacaCccuacacucucagcu..	2	1	seq
.........................guaaacauccuacacucucagcu..	25	0	seq
..........................uaaacauccuacacucucagc...	1	0	seq
..........................uaaacauccuacacucucagcu..	1	0	seq
....cugggagaggguuguuuacu.....................	19	0	seq

oar-miR-30c

```
cagacuguaaccaugccguagugugauguaaacauccuacacucucagcugugagcucgagguggcugggagaggguuguuuacuccuucugccauggaaaacguc
```
